# Real-world outcomes after switching to faricimab in previously treated neovascular age-related macular degeneration: longitudinal outcomes up to 24 months

**DOI:** 10.1186/s40942-026-00889-0

**Published:** 2026-06-23

**Authors:** Daniel Egger, Florian Amesbauer, Hamza Mohamed, Konstantin E. Thiel, Reinhard Angermann, Sebastian M. Waldstein

**Affiliations:** 1 Department of Ophthalmology, Landesklinikum Mistelbach-Gänserndorf, Mistelbach, Austria; 2 Paracelsus Medical University Salzburg, Salzburg, Austria; 3 Medical University of Vienna, Vienna, Austria; 4 Department of Ophthalmology, Tauernklinikum, Zell am See, Austria; 5 Core Facility Biostatistics, Paracelsus Medical University Salzburg, Salzburg, Austria; 6 Karl Landsteiner University of Health Sciences, Krems, Austria

**Keywords:** Faricimab, Anti-VEGF therapy, Treatment-resistance, Neovascular age-related macular degeneration

## Abstract

**Background:**

In neovascular age-related macular degeneration (nAMD), persistent disease activity despite anti-VEGF therapy is common. Switching to faricimab may improve outcomes and extend treatment intervals in treatment-resistant patients, but long-term real-world data remain limited.

**Methods:**

In this retrospective, single-centre study, 43 eyes of 38 patients with treatment-resistant nAMD were switched to faricimab and followed for up to 24 months. Insufficient disease control was defined as inability to extend treatment intervals beyond 8 weeks under a treat-and-extend regimen. Outcomes included best-corrected visual acuity (BCVA), central subfield thickness (CST), pigment epithelial detachment (PED) height, fluid status, and treatment intervals. Longitudinal changes were analysed using mixed-effects models. Exploratory analyses of baseline and early response predictors were performed.

**Results:**

Mean BCVA remained stable throughout follow-up, with no significant change from baseline at any time point (*p* > 0.05). CST decreased significantly at all visits, ranging from − 45.8 μm (95% CI − 65.3 μm to − 26.3 μm) at 1 month to − 52.3 μm (95% CI − 73.7 μm to − 30.8 μm) at 24 months (*p* < 0.001). PED height decreased at 1 month (− 19.0 μm; 95% CI − 33.6 μm to − 4.4 μm, *p* = 0.011) and remained reduced from 12 to 24 months, ranging from − 25.9 μm (95% CI − 39.7 μm to − 12.1 μm, *p* < 0.001) to − 49.1 μm (95% CI − 65.1 μm to − 33.0 μm, *p* < 0.001). Presence of subretinal fluid decreased significantly at all time points, with odds ratios (OR) ranging from 0.064 (95% CI 0.01 to 0.41, *p* = 0.004) at 1 month to 0.012 (95% CI 0.01 to 0.10, *p* < 0.001) at 24 months, whereas intraretinal fluid showed a variable response, with a significant reduction observed at 3 months only (OR 0.204, 95% CI 0.05 to 0.79, *p* = 0.021). The proportion of eyes with a dry macula increased from 0% at baseline to 23.1% at 12 months and 24.0% at 24 months. Treatment intervals extended from 5.9 ± 1.2 weeks before switch to 8.7 ± 2.6 weeks at 12 months (*p* < 0.001) and 10.6 ± 3.0 weeks at 24 months (*p* < 0.001). No adverse events were observed.

**Conclusion:**

Switching to faricimab was associated with improved anatomical outcomes and extended treatment intervals while maintaining stable visual acuity, suggesting benefits primarily related to anatomical control and reduced treatment burden.

## Background

Neovascular age-related macular degeneration (nAMD) remains a leading cause of irreversible vision loss worldwide despite substantial advances in treatment over recent decades [1]. Intravitreal anti–vascular endothelial growth factor (anti-VEGF) therapy has significantly improved visual outcomes and reduced rates of severe visual impairment. However, the need for frequent, long-term injections continues to pose a considerable burden for patients, caregivers, and healthcare systems, and real-world outcomes often fall short of those observed in clinical trials [2, 3].

Despite frequent treatment, a substantial proportion of patients exhibit persistent or recurrent exudation under ongoing anti-VEGF therapy. In clinical practice, switching to alternative agents has therefore become a widely adopted strategy to improve anatomical outcomes while maintaining or improving visual function, and potentially extending treatment intervals [4, 5]. However, long-standing disease may result in irreversible structural changes such as photoreceptor degeneration, subretinal fibrosis, and macular atrophy, which can limit the potential for visual improvement despite anatomical response to therapy [6, 7].

Faricimab, a bispecific antibody targeting both VEGF-A and angiopoietin-2, represents a novel therapeutic option in this setting [8]. By simultaneously modulating vascular permeability and stability pathways, faricimab has demonstrated improved anatomical outcomes and extended dosing intervals in pivotal clinical trials [9]. Recent systematic reviews and meta-analyses have provided pooled evidence from real-world studies supporting anatomical improvements after switching to faricimab in previously treated nAMD eyes, while functional outcomes generally remain stable over time. However, these analyses are primarily based on a limited number of studies with follow-up durations largely restricted to approximately one year and do not fully capture longitudinal real-world treatment courses beyond this timeframe [10, 11].

Therefore, this study aimed to evaluate real-world functional and anatomical outcomes after switching to faricimab in previously treated nAMD eyes, focusing on longitudinal changes over up to two years in visual acuity, retinal morphology, and treatment burden in routine clinical practice. In addition, early treatment responses and associations between baseline characteristics and longitudinal outcomes were assessed.

## Methods

### Study design

This retrospective, single-centre, longitudinal cohort study included patients with treatment-resistant nAMD receiving intravitreal anti-VEGF injections at the Department of Ophthalmology, Landesklinikum Mistelbach-Gänserndorf, Austria, between February 2023 and May 2025. The study adhered to the tenets of the Declaration of Helsinki and received approval from the Ethics Committee of Lower Austria (GS3-EK-17/063-2024).

Consecutive patients who were switched to faricimab because of insufficient disease control under prior anti-VEGF therapy were identified from electronic medical records. Insufficient disease control was defined as the inability to extend treatment intervals beyond 8 weeks within a treat-and-extend (T&E) regimen due to persistent and/or recurrent disease activity, including persistent or recurrent IRF/SRF, increased PED height, new subretinal hyperreflective material (SHRM), or haemorrhage. A minimum follow-up of 6 months after switch was required for inclusion. If both eyes of the same patient met the eligibility criteria, both were included in the analysis. Diagnosis of nAMD and exclusion of coexisting retinal diseases were based on multimodal retinal assessment, including spectral-domain OCT and dilated fundus examination as part of routine clinical care. Eyes with coexisting retinal diseases that could affect macular morphology or visual outcomes, as well as those with significant media opacity or insufficient image quality, were not eligible for inclusion.

At the time of switching, treatment strategy was determined at the discretion of the treating physician in routine clinical practice. Patients either received a loading phase of three monthly intravitreal faricimab injections followed by a T&E regimen or were directly continued on a T&E regimen using the last achieved treatment interval before switch. Treatment intervals were typically adjusted in 2-week increments based on disease activity on optical coherence tomography (OCT), with interval reduction in the presence of recurrent or persistent fluid, new subretinal hyperreflective material or haemorrhage, or an increase in pigment epithelial detachment (PED) height. Intervals were extended in the absence of disease activity. Baseline was defined as the visit at which the first faricimab injection was administered.

### Data collection

Data were collected through standardized chart review. Recorded variables included demographic characteristics such as age and sex, as well as treatment-related parameters including anti-VEGF agents before switch, treatment interval before switch, number of previous injections, post-switch treatment interval, number of injections after switch, and total follow-up duration. Best-corrected visual acuity (BCVA) measured in Snellen units was converted to logarithm of the minimum angle of resolution (logMAR) for analysis. OCT imaging was performed using a Zeiss Cirrus 6000 (macular cube 512 × 128 protocol).

Outcomes were assessed at baseline and at approximately 1, 3, 6, 12, 18, and 24 months after switching. Assessed parameters included BCVA, central subfield thickness (CST), PED height, presence of subretinal fluid (SRF) and intraretinal fluid (IRF), subretinal fibrosis defined as dense persistent SHRM, and macular atrophy.

PED height was measured as the maximum vertical distance between the retinal pigment epithelium and Bruch’s membrane within the central scan. Subretinal fibrosis was defined as dense, uniform hyperreflective material in the subretinal space. Macular atrophy was characterized by degeneration of the retinal pigment epithelium and outer retina, accompanied by choroidal hypertransmission exceeding 250 μm. Lesion location of subretinal fibrosis and macular atrophy was classified as subfoveal or extrafoveal, with extrafoveal defined as outside a 500 μm radius from the foveal centre. OCT and clinical data were independently graded by two of three masked graders (D.E., F.A., H.M.). Discrepancies were resolved by adjudication with a senior grader (S.W.).

### Statistical methods

All statistical analyses were performed using R (version 4.5.3; R Foundation for Statistical Computing, Vienna, Austria). As patients could contribute data from one or both eyes, eye-level observations were not independent. To account for the hierarchical data structure (repeated measurements nested within eyes and eyes nested within patients), longitudinal analyses were performed using mixed-effects regression models.

Changes in continuous outcomes, including BCVA, CST, PED height, and treatment interval, were analysed using linear mixed-effects models with time since switch included as a categorical fixed effect. Random intercepts for patient and eye (nested within patient) were included to account for intra-subject correlation. Binary outcomes (presence of IRF and SRF) were analysed using generalized linear mixed-effects models with a binomial distribution and logit link function. Given the limited sample size and small number of within-eye changes over time, simplified random-effects structures were applied where necessary to achieve model convergence. Macular atrophy and subretinal fibrosis were not modelled longitudinally due to absence of within‑eye temporal variation. Exploratory analyses of baseline predictors and associations between early response and long-term outcomes were performed descriptively using Pearson correlation coefficients without formal hypothesis testing. Clinically meaningful response rates (BCVA improvement ≥ 0.2 logMAR and/or CST reduction ≥ 50 μm or ≥ 10%) and the proportions of eyes achieving extended treatment intervals (≥ 8 and ≥ 12 weeks) and a dry macula defined as absence of IRF and SRF irrespective of PED persistence at 12 and 24 months were calculated descriptively. Formal hypothesis testing and reporting of p-values were limited to mixed-effects models accounting for intra-subject dependency. Continuous variables are presented as mean ± standard deviation (SD), and categorical variables are reported as counts and proportions. For binary outcomes, odds ratios < 1 indicate reduced odds of fluid presence compared with baseline. For graphical visualisation, data are presented as mean ± standard error. A two-sided p-value < 0.05 was considered statistically significant. Missing data were not imputed, and analyses were performed using all available observations.

## Results

### Demographic and baseline characteristics

A total of 43 eyes from 38 patients were included at baseline. Baseline characteristics are summarized in Table 1. The number of eyes contributing follow-up data was 33 at 1 month, 42 at 3 and 6 months, 39 at 12 months, 35 at 18 months, and 25 at 24 months, with decreasing numbers at later time points primarily reflecting limited follow-up duration rather than true attrition.

The mean age was 82.5 ± 7.3 years, and 15 patients (39.5%) were male. Patients had received a mean of 19.2 ± 12.0 anti-VEGF injections prior to switching. Before switching, most eyes were treated with bevacizumab, whereas aflibercept 2 mg was used less frequently and ranibizumab only alongside other agents. The mean treatment interval before switch was 5.9 ± 1.2 weeks, and mean follow-up duration was 85.5 ± 27.1 weeks.

Following the switch to faricimab, 31 eyes (72.1%) received a loading phase, whereas 12 eyes (27.9%) were directly continued on a treat-and-extend regimen.

At baseline, mean BCVA was 0.36 ± 0.22 logMAR, mean CST was 328.4 ± 90.8 μm, and mean PED height was 227.9 ± 122.3 μm. IRF and SRF were present in 51.2% and 65.1% of eyes, respectively. All included eyes had active exudation at the switch visit, defined by the presence of IRF and/or SRF on OCT. Macular atrophy was observed in 18.6% of eyes (25.0% extrafoveal and 75.0% subfoveal), while subretinal fibrosis was present in 11.6% of eyes, all subfoveal.

**Table 1:** Demographics and baseline characteristics at treatment switch

Study population	
Eyes, n	43
Patients, n	38
Male, n (%)	15 (39.5%)
Age, years (mean ± SD)	82.5 ± 7.3
Follow-up duration, weeks (mean ± SD)	85.5 ± 27.1
**Treatment history prior to switch**	
Total prior anti-VEGF injections, n (mean ± SD)	19.2 ± 12.0
Treatment interval before switch, weeks (mean ± SD)	5.9 ± 1.2
**Anti-VEGF agents prior to switch**,** n (%)**	
Bevacizumab only	21 (48.8%)
Bevacizumab (with other agents)	18 (41.9%)
Aflibercept 2 mg only	4 (9.3%)
Ranibizumab (with other agents)	2 (4.7%)
**Treatment regimen after switch**,** n (%)**	
Loading phase	31 (72.1%)
Direct treat-and-extend	12 (27.9%)
**Functional and anatomical characteristics**	
BCVA, logMAR (mean ± SD)	0.36 ± 0.22
CST, µm (mean ± SD)	328.4 ± 90.8
PED height, µm (mean ± SD)	227.9 ± 122.3
**OCT features at baseline**,** n (%)**	
Intraretinal fluid (IRF)	22 (51.2%)
Subretinal fluid (SRF)	28 (65.1%)
Dry macula (no SRF and IRF)	0 (0%)
Macular atrophy	8 (18.6%)
Extrafoveal	2 (25.0%)
Subfoveal	6 (75.0%)
Subretinal fibrosis	5 (11.6%)
Extrafoveal	0 (0%)
Subfoveal	5 (100%)

### Functional outcomes

**Fig. 1:**
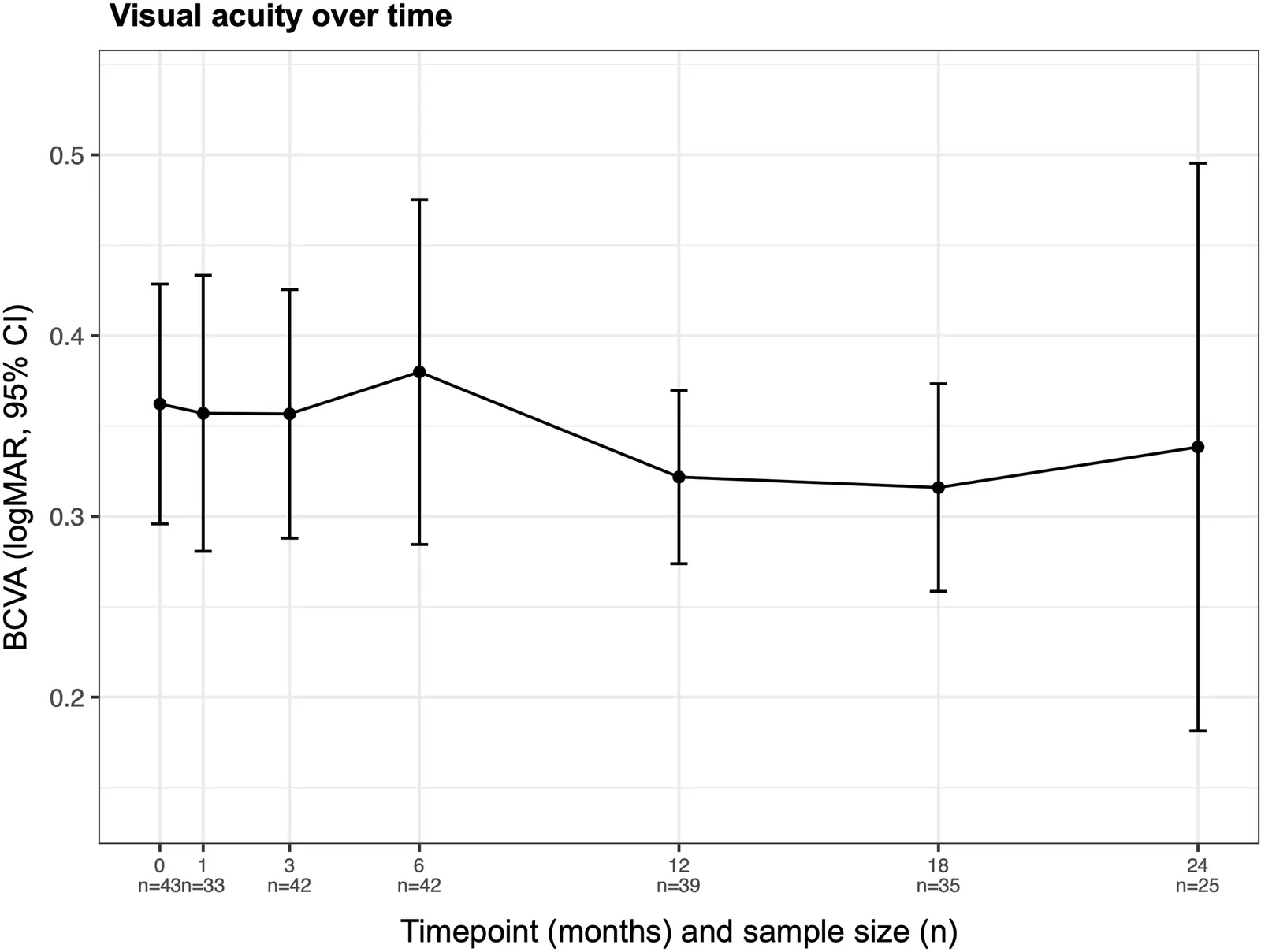
Mean best-corrected visual acuity (BCVA) over time following switch to faricimab. Error bars indicate 95% confidence intervals

Mean BCVA remained stable over time with no statistically significant changes from baseline observed at any time point, including 1 month (-0.014, 95% CI -0.08 to 0.05, *p* = 0.664), 3 months (-0.003, 95% CI -0.06 to 0.06, *p* = 0.932), 6 months (0.014, 95% CI -0.05 to 0.07, *p* = 0.636), 12 months (-0.026, 95% CI -0.09 to 0.04, *p* = 0.412), 18 months (-0.018, 95% CI -0.08 to 0.05, *p* = 0.581), and 24 months (-0.022, 95% CI -0.05 to 0.09, *p* = 0.549) (Fig. 1).

### Anatomical outcomes

**Fig. 2:**
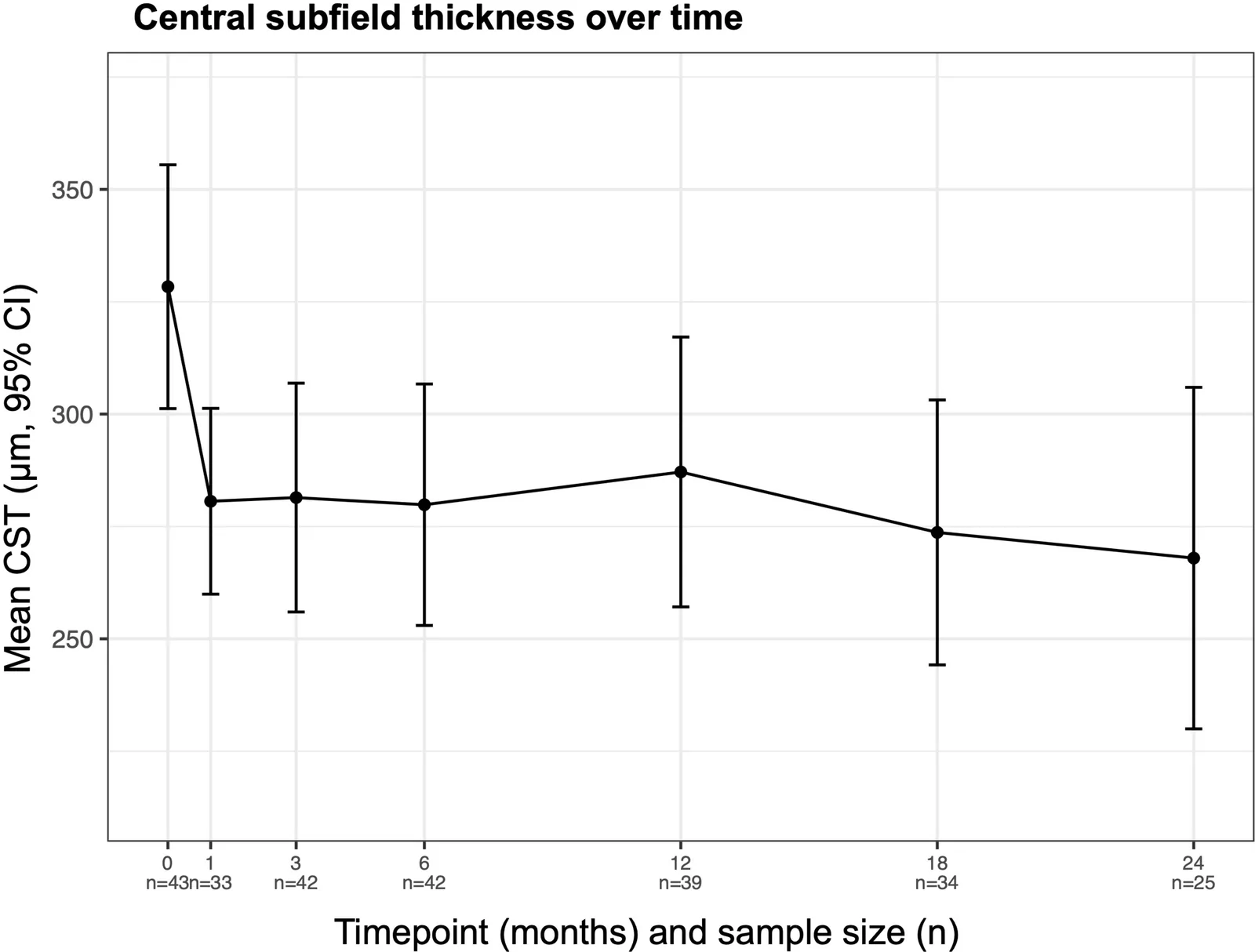
Mean central subfield thickness (CST) over time following switch to faricimab. Error bars indicate 95% confidence intervals

Mean CST showed a rapid reduction after switching, which was maintained throughout follow-up (Fig. 2). It was significantly reduced compared with baseline at all follow-up time points, including 1 month (-45.8 μm, 95% CI -65.3 μm to -26.3 μm, *p* < 0.001), 3 months (-46.5 μm, 95% CI -64.6 μm to -28.4 μm, *p* < 0.001), 6 months (-47.5 μm, 95% CI -65.6 μm to -29.5 μm, *p* < 0.001), 12 months (-41.1 μm, 95% CI -59.7 μm to -22.6 μm, *p* < 0.001), 18 months (-51.2 μm, 95% CI -70.5 μm to -31.8 μm, *p* < 0.001), and 24 months (-52.3 μm, 95% CI -73.7 μm to -30.8 μm, *p* < 0.001).

**Fig. 3:**
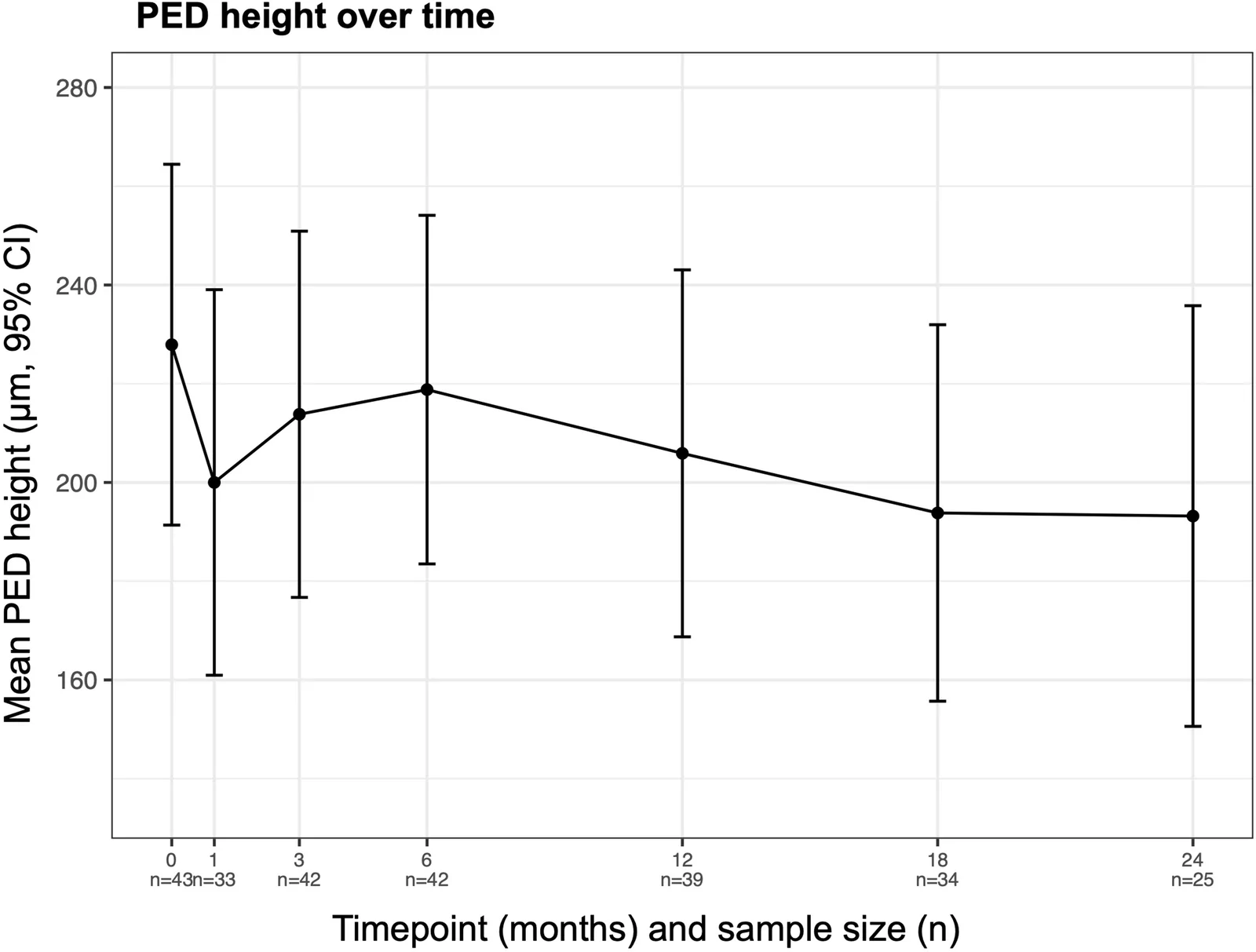
Mean pigment epithelial detachment (PED) height over time following switch to faricimab. Error bars indicate 95% confidence intervals

Mean PED height showed an initial reduction after switching, followed by minor fluctuations at early time points and a sustained decrease during longer follow-up (Fig. 3). Significant reductions compared with baseline were observed at 1 month (-19.0 μm, 95% CI -33.6 μm to -4.4 μm, *p* = 0.011), 12 months (-25.9 μm, 95% CI -39.7 μm to -12.1 μm, *p* < 0.001), 18 months (-42.1 μm, 95% CI -56.5 μm to -27.6 μm, *p* < 0.001), and 24 months (-49.1 μm, 95% CI -65.1 μm to -33.0 μm, *p* < 0.001), while no statistically significant changes were observed at 3 months (-12.8 μm, 95% CI -26.3 μm to 0.7 μm, *p* = 0.064) and 6 months (-10.4 μm, 95% CI -23.9 μm to 3.1 μm, *p* = 0.129).

**Fig. 4:**
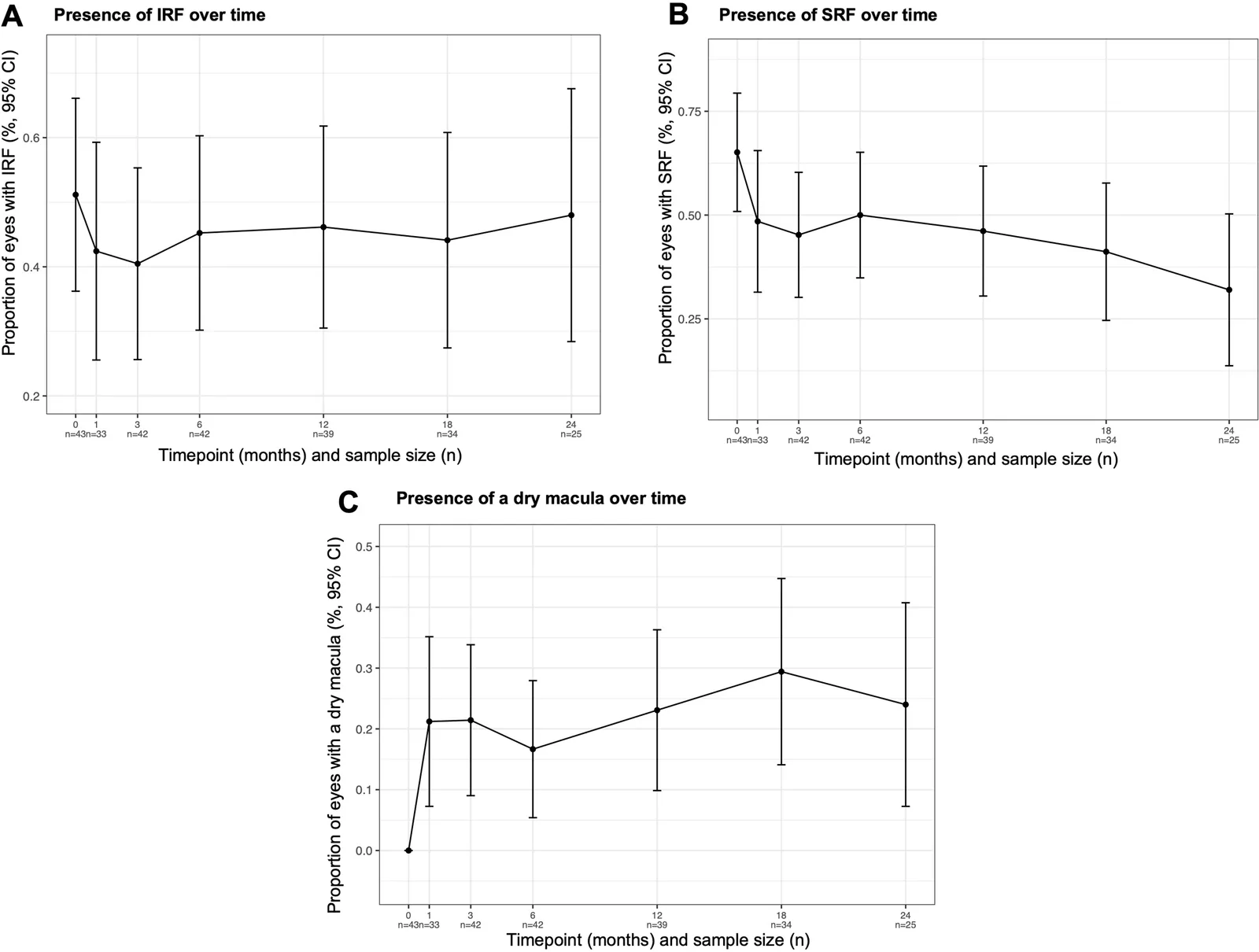
Proportion of eyes with presence of intraretinal fluid (IRF) (**A**), subretinal fluid (SRF) (**B**) or a dry macula (absence of IRF and SRF) (**C)** over time following switch to faricimab. Error bars indicate 95% confidence intervals

The proportion of eyes with IRF fluctuated over time (Fig. 4A), whereas SRF decreased consistently (Fig. 4B). In the generalized linear mixed-effects model, IRF was reduced compared with baseline at 1 month (odds ratio [OR] 0.277, 95% CI 0.07 to 1.15, *p* = 0.078), 3 months (OR 0.204, 95% CI 0.05 to 0.79, *p* = 0.021), 6 months (OR 0.516, 95% CI 0.14 to 1.92, *p* = 0.324), 12 months (OR 0.715, 95% CI 0.19 to 2.70, *p* = 0.621), 18 months (OR 0.688, 95% CI 0.17 to 2.76, *p* = 0.598), and 24 months (OR 1.597, 95% CI 0.35 to 7.19, *p* = 0.542), with a statistically significant reduction observed at 3 months only. In contrast, SRF was significantly reduced at all time points, including 1 month (OR 0.064, 95% CI 0.01 to 0.41, *p* = 0.004), 3 months (OR 0.078, 95% CI 0.01 to 0.45, *p* = 0.004), 6 months (OR 0.154, 95% CI 0.03 to 0.81, *p* = 0.028), 12 months (OR 0.086 95% CI 0.02 to 0.49, *p* = 0.006), 18 months (OR 0.051, 95% CI 0.01 to 0.32, *p* = 0.001), and 24 months (OR 0.012, 95% CI 0.01 to 0.10, *p* < 0.001). The number of eyes with a dry macula (absence of both IRF and SRF) increased following the switch to faricimab (Fig. 4C). At baseline, no eyes (0 of 43, 0.0%) presented with a dry macula. This proportion increased early to 7/33 eyes (21.2%) at 1 month and remained stable at 9/39 eyes (23.1%) at 12 months and 6/25 eyes (24.0%) at 24 months.

No new cases of macular atrophy or subretinal fibrosis and no changes in lesion location were observed during follow-up. Accordingly, no longitudinal modelling was performed for these outcomes.

**Fig. 5:**
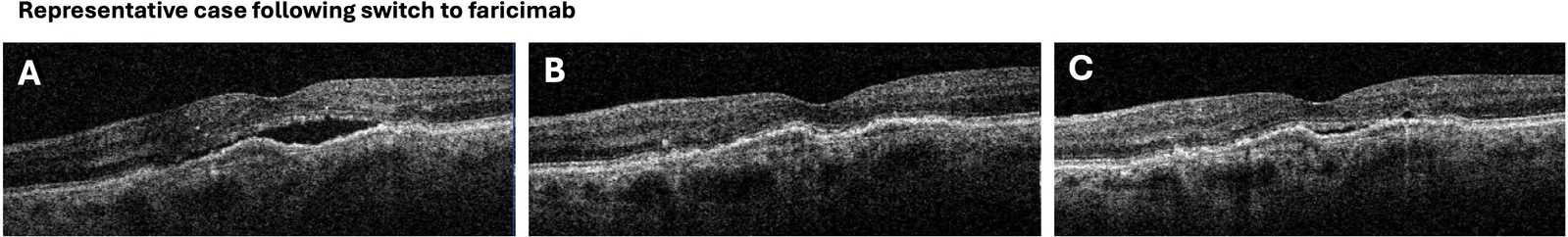
(**A**) Baseline prior to switching to faricimab after 15 previous aflibercept 2 mg injections, demonstrating persistent subretinal fluid (SRF) and pigment epithelial detachment; BCVA 0.5 logMAR, CST 308 μm, treatment interval 6 weeks. (**B**) One month after switching and following a single faricimab injection, demonstrating complete resolution of SRF and achievement of a dry macula; BCVA 0.4 logMAR, CST 193 μm. The eye subsequently received two more monthly loading injections followed by a treat-and-extend regimen and remained dry until month 24. (**C**) Twenty-four-month follow-up after extension of the treatment interval to 14 weeks, demonstrating minimal recurrent SRF; BCVA 0.4 logMAR, CST 264 μm

A representative case illustrating longitudinal anatomical changes on OCT following switch to faricimab is shown in Fig. 5.

### Treatment intervals

Treatment intervals increased significantly following the switch to faricimab. At 12 months, 61.5% (24/39) of eyes achieved intervals of ≥ 8 weeks and 17.9% (7/39) reached ≥ 12 weeks, with the mean interval extending to 8.7 ± 2.6 weeks (95% CI 7.8 to 9.2 weeks) compared with 5.9 ± 1.2 weeks at baseline (*p* < 0.001). At 24 months, these proportions increased to 92.0% (23/25) and 32.0% (8/25), respectively, accompanied by a further statistically significant extension of the mean treatment interval to 10.6 ± 3.0 weeks (95% CI 10.2 to 11.0 weeks) compared with baseline (*p* < 0.001).

### Secondary and exploratory analyses

A clinically meaningful response, defined as an improvement in BCVA of ≥ 0.2 logMAR or a reduction in CST of ≥ 50 μm, was observed in 18 of 39 eyes (46.2%) at 12 months and 11 of 25 eyes (44.0%) at 24 months. When applying an alternative definition using a CST reduction of ≥ 10%, response rates were 22 of 39 eyes (56.4%) at 12 months and 16 of 25 eyes (64.0%) at 24 months.

Associations between baseline characteristics and outcomes at 12 and 24 months were explored using Pearson correlation coefficients (r). At 12 months, BCVA was positively correlated with baseline BCVA (*r* = 0.724), CST with baseline CST (*r* = 0.533), and PED height with baseline PED height (*r* = 0.932). At 24 months, BCVA was positively correlated with baseline BCVA (*r* = 0.406) and presence of baseline IRF (*r* = 0.404), whereas baseline SRF showed a negative correlation (*r* = − 0.455). CST at 24 months was positively correlated with baseline CST (*r* = 0.663), and PED height at 24 months with baseline PED height (*r* = 0.912).

Additionally, associations between early changes in BCVA and CST at 1 and 3 months and outcomes at 12 and 24 months were also explored using Pearson correlation coefficients. Greater early CST reduction at 1 month was moderately associated with better visual acuity at 12 months (*r* = − 0.481), while other associations were negligible.

### Safety

No ocular or systemic adverse events were observed during follow-up, including no cases of intraocular inflammation, retinal vasculitis, endophthalmitis, or retinal detachment.

## Discussion

In this real-world cohort of treatment-resistant nAMD patients, switching to faricimab resulted in anatomical improvements over 24 months, accompanied by a progressive extension of treatment intervals from 5.9 to 10.6 weeks, while visual acuity remained stable. Together, these findings suggest improved anatomical control with a reduction in treatment burden. Importantly, the present study extends existing evidence by providing longitudinal real-world outcomes up to 24 months in a chronically treated cohort with persistent disease activity despite prior anti-VEGF therapy.

The observed anatomical response was consistent with other real-world studies investigating switch to faricimab in eyes with previously treated nAMD [12–15]. CST decreased significantly at all time points, and PED height showed a sustained reduction from 12 months onwards. Exploratory analyses suggested a possible association between early anatomical response and later outcomes. Greater CST reduction at 1 month was moderately associated with better BCVA at 12 months (*r* = − 0.481), indicating that early treatment response may serve as a marker of subsequent functional outcomes. These findings should be interpreted with caution given their moderate association and exploratory nature but are consistent with prior reports demonstrating rapid anatomical improvements after faricimab initiation, suggesting early treatment sensitivity [16–18].

A notable finding of this study is the divergent response between SRF and IRF. While SRF decreased consistently, IRF showed a less pronounced and more variable response. This pattern suggests distinct underlying pathophysiological mechanisms, with persistent IRF potentially representing more advanced or chronic retinal damage [19–21]. In addition, these findings may reflect differential sensitivity of retinal fluid compartments to initial anti-VEGF therapy, with IRF being the most responsive and SRF and PED the least. Consequently, IRF may already be maximally suppressed under prior anti-VEGF therapy in many chronically treated eyes, potentially limiting further improvement after switching, whereas SRF and PED may remain more responsive to treatment changes [22]. The dual inhibition of VEGF and Ang-2 by faricimab may enhance vascular stability and promote fluid resorption, potentially exerting a preferential effect on subretinal compartments. This is supported by a post-hoc analysis of TENAYA/LUCERNE, which demonstrated higher rates of SRF resolution with faricimab compared to aflibercept (87.9% vs. 79.0% at week 12), whereas IRF outcomes were largely comparable, suggesting a more pronounced effect on SRF [23].

At baseline, no eyes were free of both IRF and SRF in this study, whereas approximately one quarter of eyes achieved a dry macula at 12 and 24 months. Notably, this effect was evident as early as 1 month, with over one-fifth of eyes achieving a dry macula after the first faricimab injection, indicating a rapid initial drying response that was sustained over long-term follow-up. However, the proportion of eyes achieving a dry macula in our cohort was lower than previously reported, with Janmohamed et al. [15] describing rates of 40.2% and Sim et al. [13] 33.7% at 12 months. This difference may be explained by variations in baseline morphology, as Sim et al. [13] reported a proportion of eyes with a dry macula already at baseline (18.3%), as well as differences in treatment strategies and retreatment criteria, which are known to influence fluid outcomes in real-world studies [24].

Despite these anatomical changes, BCVA remained stable, with no significant change from baseline at any time point. The lack of functional improvement despite clear anatomical benefits is a consistent finding in switch populations and likely reflects the advanced disease stage of these patients. Eyes in our cohort had received a mean of 19.2 ± 12.0 prior anti-VEGF injections, indicating long-standing disease. The limited functional improvement despite anatomical drying may partly reflect irreversible photoreceptor and retinal structural damage (i.e. macular atrophy and subretinal fibrosis) in this chronically treated population [6, 7, 25]. However, despite stable mean BCVA at the cohort level, nearly half of eyes achieved a clinically meaningful functional and/or anatomical response at 12 and 24 months. In line with our findings, two recent meta-analyses demonstrated no significant change in BCVA following switching to faricimab when aggregating data across studies [10, 11]. However, individual real-world studies, including those by Rush [12], Janmohamed et al. [15], and Khodor et al. [26], reported statistically significant improvements in BCVA at 12 months. This discrepancy likely reflects differences in cohort characteristics, including baseline BCVA and disease chronicity.

A key clinical finding of this study is the progressive extension of treatment intervals over time. At 12 months, 61.5% of eyes achieved intervals of ≥ 8 weeks and 17.9% reached ≥ 12 weeks, increasing to 92.0% and 32.0% at 24 months, respectively. These findings are broadly consistent with prior 12-month real-world studies. Ambati et al. [27] reported an increase from 5.9 to 7.6 weeks, while Janmohamed et al. [15] observed a rise from 4 to 8 weeks, and Sim et al. [13] reported a mean interval of 6.9 weeks at 12 months, with 42.9% achieving ≥ 8-week intervals. Importantly, these interval extensions translate directly into reduced treatment burden and clinic visits for patients while alleviating demands on caregivers and healthcare systems [28, 29]. Given the chronic nature of nAMD, this has relevant socioeconomic implications, including improved patient convenience and potential gains in adherence [30]. Notably, our results further suggest that with longer-term treatment over 24 months, treatment intervals may continue to extend beyond those reported at 12 months, indicating a progressive reduction in treatment burden over time.

Alongside these improvements in treatment burden, structural stability and safety remain critical in long-term management of nAMD. There were no new cases of macular atrophy or subretinal fibrosis observed during follow-up, indicating structural stability in this cohort. Furthermore, no unexpected safety signals were identified, supporting a favourable safety profile of faricimab in this real-world setting.

This study has several limitations. Its retrospective, single-centre design may limit generalizability and the absence of a control group receiving continued prior anti-VEGF therapy limits causal interpretation of the observed outcomes. In addition, switching decisions were made at the discretion of the treating physician, which may have introduced variability in patient selection. Patients received either a loading phase or direct continuation of a T&E regimen after switching, which may have contributed to heterogeneity in early treatment responses. Prior anti-VEGF exposure was heterogeneous, including bevacizumab, aflibercept, ranibizumab, and combination treatment histories, which may have influenced treatment response after switching. Notably, the proportion of aflibercept-pretreated eyes was relatively low compared with most previously published refractory switch cohorts, which should be considered when comparing outcomes across studies. Additionally, the operational definition used in this study reflects limited treatment durability within a T&E regimen rather than proven biological anti-VEGF resistance in all cases.

The decreasing number of eyes available at later time points, particularly at 24 months, primarily reflected the timing of faricimab initiation relative to the predefined study cutoff rather than true attrition or treatment discontinuation. Nevertheless, the smaller sample size at later visits limits the robustness and interpretation of long-term outcomes. Accordingly, several analyses were exploratory in nature, and formal hypothesis testing was limited to the prespecified mixed-effects models. Model diagnostics indicate some limitations, including outliers and residual irregularities (such as skewness) for parts of the continuous outcomes, and simplified random-effects structures for some models. The results are therefore informative, but should be interpreted with appropriate caution.

In conclusion, switching to faricimab in treatment-resistant nAMD resulted in sustained reductions in CST, PED height, and SRF, with stable BCVA and a marked extension of treatment intervals. These findings support faricimab as an effective option in this patient population, with clinical benefits primarily driven by improved anatomical control and reduced treatment burden rather than functional gain.

## Data Availability

The data are available from the corresponding author upon reasonable request.

## Abbreviations

VEGF: Vascular endothelial growth factor
Ang-2: Angiopoietin-2
nAMD: Neovascular age-related macular degeneration
BCVA: Best-corrected visual acuity
CST: Central subfield thickness
PED: Pigment epithelial detachment
IRF: Intraretinal fluid
SRF: Subretinal fluid
OCT: Optical coherence tomography
T&E: Treat-and-extend
logMAR: Logarithm of the minimum angle of resolution
OR: Odds ratio
SD: Standard deviation
RPE: Retinal pigment epithelium
